# “I Can’t Do without You”: Treatment Perspectives for Affective Dependence: A Scoping Review

**DOI:** 10.3390/jcm12216769

**Published:** 2023-10-26

**Authors:** Zeynep Özal, Giacomo Mancini, Greta De Fino, Federica Ambrosini, Roberta Biolcati, Roberto Truzoli

**Affiliations:** 1 Department of Education Sciences “G.M. Bertin”, Alma Mater Studiorum, University of Bologna, 40126 Bologna, Italy; zzeynepozal@gmail.com (Z.Ö.); federica.ambrosini3@unibo.it (F.A.); r.biolcati@unibo.it (R.B.); 2 Department of Biomedical and Clinical Sciences, University of Milan, 20157 Milano, Italy; greta.defino@unimi.it (G.D.F.); roberto.truzoli@unimi.it (R.T.)

**Keywords:** affective dependence, love addiction, pathological love, emotional dependence, relationship addiction, treatment, clinical interventions

## Abstract

Affective Dependence (AD) is a problematic model of the love relationship that is becoming increasingly prevalent and evident in the context of couple relationships. Similarly, the phenomenon is being observed with growing frequency in daily clinical practice, making it increasingly necessary to identify treatment guidelines that can help clinicians in dealing with AD, while waiting for the literature to reach a consensus on its definition and nosographic profile. The main objective of this work is to explore the existing evidence of effectiveness regarding feasible treatments for Affective Dependence through a scoping review of the international literature carried out using the main scientific databases and following the PRISMA-ScR (PRISMA Extension for Scoping Reviews) guidelines. Seven studies were included in this review, and the results show that several pharmaceuticals, as well as different types of individual and group therapies, are proposed as treatment plans for AD. However, there is a lack of clinical trials that can verify the efficacy of the AD treatments reported so far in the literature. Some reflections that may help to distinguish a “healthy” addiction from a dysfunctional or markedly pathological one are considered alongside potential prevention perspectives.

## 1. Introduction

As difficult as it is to accept, even love, which is one of the most valued and appreciated human experiences, can lead to psychopathology. Although there is not a strictly recognized framework for Affective Dependence (AD)—also termed love addiction (LA)—there have been suggestions for its definition. The absence of reciprocity within a romantic relationship [1], relational distortion as a form of morbid, obsessive, and fusional love that is detrimental to a person’s well-being [2], and a compulsive need for closeness that results in dysfunctional relational patterns [3] are present in some of the proposed definitions. The person suffering from LA builds his or her existential project entirely around the couple’s relationship, which is seen as a drug that one cannot do without. This relationship fills one’s emotional voids as the only source of satisfaction, even if the individual no longer receives love from it. The other person becomes so important that one cancels oneself out, does not listen to his or her own needs, and sacrifices any personal evolutionary impulse [4]. Therefore, the love relationship, instead of being an opportunity for growth, becomes a symbiotic and stagnant condition: in most cases unsatisfactory, unhappy, painful, and a source of frustration and dissatisfaction. AD is usually established between two adult partners, but it can also occur in family relationships or even in the therapeutic relationship between a clinician and a patient [2]. For this reason, it is important for the clinician to prevent the therapeutic relationship from drifting in a direction completely contrary to its intended goal, and to facilitate the patient’s autonomous growth. It is essential to establish boundaries (i.e., time, space, and the rules that define the therapeutic relationship) between the therapist and the client to prevent the risk of their therapeutic relationship developing in an unintended direction.

The first valid systematic set of criteria for LA was proposed by Michel Reynaud and colleagues [3]. Some of these criteria include an excessive amount of time spent on the relationship and, as a result, disengagement from social and work life, as well as attachment problems. In brief, a phenomenological framing of AD in relationships may have the following characteristics: fear of being abandoned and a need for control over the relationship; inability to cope with loneliness and difficulties with separation [5]; emotion dysregulation [6]; feeling undeserving of love as a result of low self-esteem and self-confidence [7]; complete dedication to the partner and total acceptance [8]; and a tendency towards isolation and obsessive thoughts [9].

Although it is a widespread form of bonding in modern society, the concept of AD, in itself, has roots that extend far back in history. However, it is only in recent times that we have witnessed growing interest and increased research regarding this condition, particularly since the early 1980s. Nevertheless, despite the increasing number of publications, existing empirical research on this phenomenon remains scarce, and statistical data seem to be almost non-existent. In addition, there are no officially recognized criteria in the *Diagnostic and Statistical Manual of Mental Disorders Fifth Edition* (DSM-5) [10] that could be the basis for the clinical diagnosis of AD. However, AD is considered part of the so-called ‘New Addictions’ or ‘Behavioural Addictions’, which were introduced into the official classification for the first time with the DSM-5. Nevertheless, there is still no consensus on the theoretical basis of AD. There are different views regarding whether AD should be based on Griffiths’ component model [11,12]; it would be either a consequence of specific attachment processes [13]; it would be highly correlated with particular personality traits [14]; or it may not be considered as a behavioural disorder because it is a form of personality disorder [15]. 

In some ways, subjects suffering from AD share part of their symptomatology with other psychopathological conditions such as obsessive-compulsive disorder (OCD) [16], impulse-control disorder (ICD) [14], and dependent personality disorder (DPD) [17] because they can co-occur. However, despite a possible co-occurrence, different conditions like AD and DPD are not exactly comparable. For instance, in DPD, the person on whom one is dependent can be substituted by another, whereas in AD the subject shows a tendency to remain anchored in a particular relationship, trying in every way to regain it. It should be noted that a state of AD is not necessarily indicative of a DPD; rather, it emphasizes the need to define and frame such dependency more precisely, within a specific individual profile.

As is the case with most psychopathological manifestations, the causes of AD can be traced back to childhood. Indeed, several studies in the literature clearly show the role of primary attachment experiences in the aetiology and development of this disorder [2,18,19,20,21]. As a result of negative emotional experiences with caregivers (e.g., parents, guardians, or fosters), the child may begin to believe that he or she is not worthy of love and that his or her own needs are unimportant. This can lead to anxiety, guilt, emotional instability, or fear of abandonment, separation, and loss [22]. The intrinsic belief that one is not worthy of love also inevitably influences the choice of partner and the type of relationship experienced. It is not uncommon for the affective dependent, in his or her intermittent search for love, to relate to partners who are egocentric, ineffective, rejecting or avoidant, elusive or unattainable (e.g., a person disinterested in the relationship or engaged in another relationship), and who do not care about the well-being of the dependent. Furthermore, it is not surprising that the affective addict tends to relate to individuals who exhibit typical Cluster B personality traits (e.g., borderline, narcissistic, and antisocial).

Although an analysis of epidemiological data shows that AD appears to be a predominantly female pathology [23], there are cases in men as well, and it has different characteristics and behavioural manifestations (e.g., attitudes of control, dominance, emotional manipulation of the partner, offence, or use of violence) [5]. Moreover, the same dynamics can also be found within homosexual couples [24]. Although the couple relationship is the context in which AD most often occurs, this condition can develop in any relationship that is significant in a person’s life (e.g., a parent, another family member, a friend, or a person of authority) [4]. 

However, prevalence estimates of AD in the general population are still unclear; this is partly due to the scarcity of validated psychometric instruments in the literature [25]. The few that are mentioned in the literature are as follows: Love Scale (LS) [26], Love Attitudes Scale (LAS) [27,28], Peabody’s Addiction to Love Questionnaire (PALQ) [16], Love Addiction Inventory (LAI) [12], and Affective Dependence Scale (ADS-9) [29]. All of the instruments are self-administered scales and only two of them [12,29] have validation studies. Furthermore, these instruments measure AD by relying on different theoretical models. For instance, while LAI is developed based on the component’s model [11,12], ADS-9 is based on two factors, craving and submission [29]. A review carried out by Maglia and colleagues presents findings on the use of some of these measures, particularly the LAS and the LAI [30]; another study also aimed to guide research on the assessment of LA [6]. In addition to these scales, further instruments for AD symptom self-assessment are available on the Internet; the best-known scale is the self-administered, 40-question test of Sex and Love Addicts Anonymous (SLAA) [31], which is designed to be a preliminary self-diagnosis tool to assess possible adherence to meetings organized by the association, and Love Addiction Self-Assessment (LASA) [32], which is adapted from the SLAA tool.

In summary, Affective Dependence seems to represent a distressing condition that is now widespread in contemporary romantic relationships, and requests for its treatment in clinical settings are increasing. Yet, at present, it is rather complex for clinicians to establish a treatment plan in daily practice. This work, through a scoping review of the scientific literature, aims to provide an update on how current research informs clinicians regarding the treatment of individuals with AD and aims to systematically map the research conducted in this area, identifying existing gaps in the knowledge. 

## 2. Materials and Methods

The reporting of this scoping review followed the PRISMA-ScR (PRISMA Extension for Scoping Reviews) guidelines [33].

### 2.1. Information Sources

Searches were performed in Google Scholar, PubMed, Scopus, Web of Science, PsycINFO, and Cochrane Library in June 2023 using the following keywords: *Affective Dependence*, *Love Addiction*, *Pathological Love*, *Emotional Dependence*, and *Relationship Addiction*.

### 2.2. Eligibility Criteria

The eligibility criteria for the search strategy and study selection were strictly defined prior to the search. The inclusion criteria for the search strategy were articles published (a) in English, (b) as journal articles, and (c) between the years 2000 and 2023. The inclusion criterion for study selection was having treatment perspectives for AD/LA as the article topic.

Any search result that was in a format other than a scientific article (e.g., conference paper, book chapter, or master’s thesis), published in a language other than English, or had a different subject focus and was published before 2000 was excluded.

### 2.3. Search Strategy and Study Selection

In the selected databases, in a single search string, all the aforementioned keywords were enclosed in quotation marks and separated by the Boolean operator *OR*. The databases were set to display records including the keywords in the title, abstract, and keywords sections of the papers. The year range and publication language were set to 2000–2023 and English, respectively. The document type was either set differently in each database due to the diverse options they provided or was not set because of the absence of a specification option. Accordingly, “*Only articles*”, “*Articles & Reviews*”, and “*All Journals*” were selected for Web of Science, Scopus, and PsycINFO, respectively, but no options were selected for the remaining databases.

### 2.4. Data Extraction

**Figure 1:**
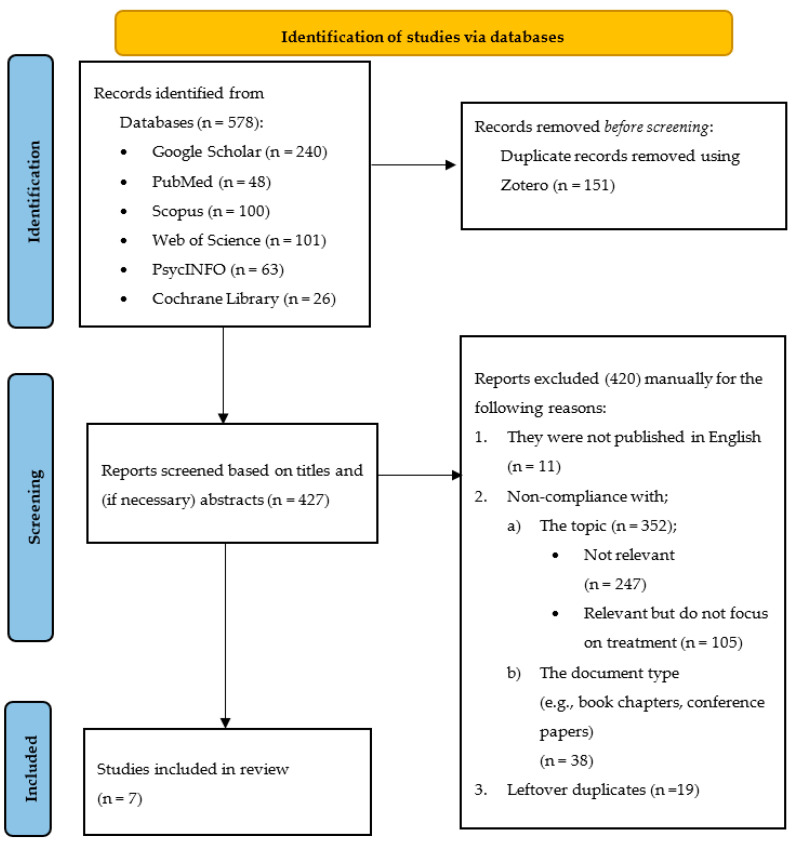
PRISMA flow diagram of the study selection process.

After the identification of search results, a further evaluation based on search and selection criteria was carried out. Firstly, 151 duplicate records were eliminated using a reference management software called *Zotero 6.0.27* [34]. Then, titles were screened to eliminate the many unrelated search results, as well as studies not meeting the eligibility criteria. In cases where additional information was needed for screening, abstracts were also checked. As a result of the screening process, a total of 7 articles were included in the review. The data extraction process is summarized in the flow diagram in Figure 1.

### 2.5. Organization of the Results

The results of the included articles are summarized in Table 1 and organized under 4 main subtitles: (1) Individual Psychotherapy (*Cognitive Behavioural Therapy*, *Psychodynamic Psychotherapy*, *Gestalt Therapy*, *Integrated Psychotherapy: Imago Therapy and Schema Therapy*); (2) Group Psychotherapy (*Psychodrama Group Therapy*, *Self-Help Groups*); (3) “Healthy Replacement Reinforcers”; and (4) Pharmacological Interventions.

## 3. Results

### 3.1. Individual Psychotherapy

#### 3.1.1. Cognitive Behavioural Therapy

Cognitive Behavioural Therapy (CBT) has been cited as a potentially effective intervention for the treatment of AD [38,40]. Indeed, many of the psychopathological features of AD seem to stem from automatic and dysfunctional thought processes [4,22]. Such automatisms could be identified and successfully addressed through CBT, which would also be effective in improving self-communication and the separation between emotions, thoughts, and actions [40]. In addition, CBT has often proven effective in managing emotional dysregulation, which is also described as one of the central aspects of AD. Indeed, the development of good emotional skills is essential for preventing relapse. However, it was not possible to identify studies confirming the efficacy of CBT in the treatment of Affective Dependence.

#### 3.1.2. Psychodynamic Psychotherapy

As previously mentioned, it has been hypothesized that AD can occur as a result of attachment experiences, which can be traced back to early childhood. Therefore, some authors claim that psychodynamic psychotherapy would be a promising therapeutic approach for the treatment of AD [4,23]. In this case, early childhood experiences and dysfunctional attachment patterns would be the primary objects of analysis, guiding the person towards a better understanding of the origins of his or her own sense of abandonment. Again, however, studies investigating its effectiveness could not be found. It has also been suggested that the psychodynamic treatment of LA may be quite complex because the relational difficulties of patients (e.g., attachment and intimacy issues) could lead to problematic transference and counter-transference reactions [40].

#### 3.1.3. Gestalt Therapy

Gestalt Therapy (GT), which focuses on the present awareness and the interaction between the individual and the environment, is proposed as another treatment plan for LA. As mentioned earlier, people with LA may experience low self-esteem, feelings of being unworthy of love, a fear of abandonment, and difficulties in setting boundaries regarding intimacy by invading the personal space of the partner on whom they depend [17]. According to Raffagnino and Zerbetto [41], clinicians with GT orientation can aim to complete three steps as follows: restore the sense of self-regulation of the clients with LA, build their capacity for self-acceptance, and build more balanced relationships. While focusing on these goals, the key element in the therapeutic process is the relationship between the therapist and the client. Despite the emphasis on the here-and-now, in GT, it is also suggested to address unresolved issues stemming from the past [41]. Considering the case vignettes presented in the above-mentioned article [41], GT appears to be a treatment option for LA. However, there is no scientific evidence demonstrating the effectiveness of GT in the treatment of LA.

#### 3.1.4. Integrated Psychotherapy: Imago Therapy and Schema Therapy

Among the reviewed literature, a semi-experimental study [39] aimed to investigate the effectiveness of an integrative approach, consisting of Imago Therapy and Schema Therapy, as a possible treatment plan for LA. A total of 30 girls, divided into an experimental group and a control group, were administered the Peabody’s Addiction to Love Questionnaire (PALQ) twice (i.e., before the treatment initiation and after the experimental group had completed all therapy sessions). In addition to a single two-hour group session, the experimental group received weekly sessions for one month, for a total of about eight hours. While the Imago Therapy approach is used to explore the childhood sources of LA and early attachment experiences, the Schema Therapy approach is adapted to define ineffective schemas and modes for replacing unhealthy dependencies with effective coping mechanisms [39]. The results show that, compared to the control group, a significant reduction in LA is observed in the experimental group as a result of the integrated psychotherapy sessions. However, these results are not sufficient to formulate conclusive recommendations regarding the application of such integrated therapy for the treatment of LA.

### 3.2. Group Psychotherapy

#### 3.2.1. Psychodrama Group Therapy

Psychodrama group therapy has proven to be an effective approach, helping to develop insight into interpersonal relationships and improve self-esteem [30]. For this reason, Lorena and colleagues [35] conducted a study in which eight subjects with a presumed diagnosis of pathological love underwent 18 weekly sessions of psychodrama group therapy. The Love Attitudes Scale (LAS) was used as the measurement tool. The results showed that five out of eight participants showed significant improvement as a result of 18 weeks of therapy [35]. Although psychodrama group therapy is a promising approach, the small sample size and lack of a control group make it difficult to generalize.

#### 3.2.2. Self-Help Groups

Self-help groups are one of the most widely used psychosocial interventions in the treatment of LA; most of these are based on the 12-step principle [40]. Group interventions are fundamental to breaking out of isolation and experiencing new, healthier, and more functional forms of relationality, as well as restoring a satisfying social-relational context. In the United States, the best-known group is Sex and Love Addicts Anonymous (SLAA). However, despite the popularity of self-help groups and the fact that they are often cited by experts as a pillar in the treatment of LA, no controlled studies evaluating their efficacy have been published.

### 3.3. Healthy Replacement Reinforcers

In addition to the several types of therapies described so far, individual-level treatment options were also considered. In most cases, these are purely behavioural strategies. Fisher and colleagues [37] argue that abstinence from the object of desire (e.g., removing photos, cards, and songs and avoiding contact with the rejecting partner) is a fundamental aspect of recovery from affective addiction. This is because the memories and contact with the partner can act as triggers, causing cravings and interfering with the healing process [37]. When cravings and intrusive thoughts become more intense, the subjects must commit to waiting and resisting by trying to redirect their thoughts and behaviour [42]. In order to alleviate AD symptoms, it is essential for the person to be exposed to new environments to facilitate healthier and more positive experiences. For instance, developing new daily habits, meeting new people, and engaging in new and stimulating activities (e.g., hobbies, sports, volunteering, spiritual experiences, meditation, etc.) can provide a rewarding substitute and distraction, precisely because of their potential to generate positive feelings and activate reward mechanisms [37].

### 3.4. Pharmacological Interventions

Regarding the pharmacological treatment of AD, there are no specific and validated treatment guidelines in the literature to recommend any interventions in clinical practice [36]. However, based on the presumed neurobiological mechanisms involved in the development of AD and its phenomenological characteristics, it can be hypothesized that certain classes of psychopharmacological molecules might have a potential role in the management of AD [17]; therefore, it is worth exploring the possible options. It has been suggested that antidepressants and mood stabilizers can be useful as they improve mood dysregulation [36,38,40]. For example, on the basis of a partial symptomatologic (both cognitive and neurochemical) overlap between LA and obsessive-compulsive disorder (OCD), treatment with selective serotonin reuptake inhibitors (SSRIs) has been proposed [40]. As SSRIs appear to reduce dopaminergic transmission in the ventral tegmental area, where some of the reward and motivation areas are located [43], they may have a therapeutic role in the treatment of patients with LA. Furthermore, the strong association between AD, impulsivity, and emotional dysregulation suggests that treatment with mood stabilizers, such as lithium, which has proven effective in treating aggression and other behaviours secondary to poor impulse control, could also be considered [36,40]. Again, given the presumed association between dopaminergic activity and romantic feelings, it might be hypothesized that antipsychotics may play a role in the treatment of AD due to their antagonistic effect on the dopaminergic system [40]. Finally, considering the phenomenological features shared by affective and substance dependence (e.g., craving, tolerance, and withdrawal phenomena), it could be hypothesized that pharmacological agents that are used to treat other addictions, such as naltrexone and buprenorphine, might also prove effective in the treatment of this condition [32]. However, it is important to reiterate that, to date, there are no scientific data available on the efficacy, let alone the safety, of pharmacological interventions in the treatment of AD. Consequently, precisely because of the lack of available scientific data, pharmacological interventions are not a recommended treatment based on evidence of efficacy. It should also be emphasized that the use of medications specifically targeting AD symptoms could raise important bioethical concerns [40].

## 4. Discussion

The present scoping review of the literature summarizes how clinicians address and attempt to treat Affective Dependence. Through the detailed identification and screening of the available search results, seven articles were included in this review. The results of this review indicate that, although this condition has long been a topic of discussion and interest, scientific evidence regarding its treatment is still limited. It could be concluded that first-line treatment for AD, as well as for other forms of addiction, should be sought among the so-called ‘traditional’ therapies, such as CBT, different kinds of group therapies, psychoanalysis, or even a combination of these treatment modalities, acting mainly at the psycho-behavioural level [35,37,38,39,40,41,44]. Self-help groups such as SLAA or healthy new habits like hobbies, sports, volunteering, spiritual experiences, and abstinence from the object of desire (i.e., the partner whom one is dependent on or anything that reminds him/her) are also suggested [37,40]. 

It is complicated to determine if a form of love is the regular ‘falling in love’ type or a pathological one; it must be noted that there are also ethical considerations while making such a distinction [38]. In fact, it could be argued that considering an addiction to love as a pathological manifestation, or even just studying love from a scientific perspective, could somehow deprive it of its value and importance, reducing it to an anonymous set of chemical and neurobiological reactions. It is, therefore, considered essential to try to delineate a boundary between what can still be considered healthy attachment and what represents a more markedly pathological extension of love addiction [45,46]. Yet, given the commonalities between AD and other addictions and behavioural disorders, several studies also suggest the possibility of following pharmacological treatment plans [36,38,40]. In particular, antidepressants and mood stabilizers have been cited as potentially useful in helping to regulate aggression and impulse control. However, no scientific evidence is available to support either the effect of the aforementioned therapies and suggested substitutive habits or the use of pharmacological agents in the treatment of AD.

### 4.1. Pathological Limits of Addiction in Love

It is also stated that there might not be a need for treatment, of course, if this dependence is not harmful [38]. From the perspective of Fisher and co-workers [36], the romantic relationship is characterized as a ‘positive’ dependency if it is reciprocal, balanced, and a source of well-being for the couple, and ‘negative’ and harmful if it is one-sided, inappropriate, or formally rejected. According to Griffiths, “the difference between an excessive healthy enthusiasm and an addiction is that healthy enthusiasms add to life, whereas addictions take away from it” [11] (p. 195). As already mentioned, the difference between AD and a healthy addiction to love is not always so clear-cut, and it can sometimes happen that the two phenomena are confused. It is highlighted that ‘romantic love’ corresponds to a specific pattern of physiological, psychological, and behavioural characteristics that can be likened to the characteristic symptoms of other addiction disorders. These include, for example, the presence of energizing and euphoric feelings, mood swings, compulsive desire (e.g., craving), a cognitive process characterized by object-focused attention and pervasive and recurring thoughts, tolerance, an intense motivation to establish and maintain a bond, affiliative behaviours, the reorganization of one’s time, space, and priorities according to the other, and, finally, emotional and physical dependence and need for the other [37,42].

### 4.2. Is It Possible to Prevent?

As in many types of addiction, AD suffering and dedication to one’s partner can reach extremes. For example, it may seriously compromise an individual’s physical and psychological safety; in the most extreme cases, in fact, the victim may even tolerate exploitation, manipulation, abuse, or violence, which may be verbal or even physical. These situations can have dramatic consequences, often leading to criminal events such as crimes of passion, suicides, and murder–suicides [46]. Therefore, it seems urgent and absolutely necessary to implement preventive strategies for this disorder, which can potentially be a very serious condition, in order to prevent dramatic consequences.

There are numerous potentially effective prevention strategies for the development of AD, both at the individual and collective level. Individual strategies can be summarized as follows: counseling interventions [38] aimed at developing a more secure attachment style and developing self-esteem; emotion management techniques in order to counteract one of the main reasons for seeking a partner; emotional skill learning [18]; and certain cognitive restructuring techniques to prevent the role of automatic thoughts and cognitive distortions in the cause and development of the disorder. Given the influence of the family and the cultural context in one’s developmental history [8], psycho-educational interventions for romantic relationships can be considered. On the basis of these considerations, in addition to the individual prevention strategies outlined so far, consideration is also given to collective prevention planning to promote healthier relationship patterns. Some of the collective-level prevention strategies could involve the mass media, with programmes to disseminate corrective information on the distinction between healthy and dysfunctional romantic relationships; in the school environment, to provide education on healthy relationships as part of school curricula; or in the general population, through awareness campaigns [19]. It would, undoubtedly, be effective to implement strategies that disconfirm certain myths and ideologies about the ideal of romantic love: for instance, the myth that there is only one ‘right person’ for each of us, or the myth that being obsessed with another person is a sign of love. Lastly, some political actions could also be considered for preventive purposes, such as the publishing of warning statements in major media outlets that deal with the dissemination of material related to romantic relationships.

## 5. Limitations

Our (scoping) review has a number of limitations. Firstly, our search was limited to the databases in which we conducted our search and was subject to language bias as we only included studies published in English; also, the exclusion of grey literature may have resulted in the loss of some information. Lastly, protocol registration is not available for this review. 

## 6. Future Directions

There are a number of points that need to be addressed in future research. First of all, establishing a shared definition of Affective Dependence (i.e., what it is and what it is not) is necessary; related terminology should also be clarified as there are several terms that are used interchangeably (e.g., pathological love, love addiction, emotional dependency, affective dependency, emotional addiction, and relationship addiction). The absence of a consensus on definition and terminology also puts clinicians at risk in terms of how to interpret certain symptoms and plan interventions. In addition, perhaps also because of the lack of officially recognized criteria for AD, designing clinical trials to investigate the effectiveness of intervention strategies has been discouraged in the literature. This is, unfortunately, obvious considering the very few search results we obtained, as shown in Table 1. Furthermore, it is also necessary to work on measurement methods for AD, as well as their validated versions in different languages and cultures.

## 7. Conclusions

Affective Dependence (AD), as a problematic model of love, has become increasingly common both in couple relationships and in daily clinical practice. This scoping review has focused on how current research informs clinicians on the treatment of AD. The results of seven studies showed that different pharmacological (e.g., SSRIs and mood stabilizers) and psychotherapeutic (e.g., different types of individual and group therapies) treatment plans for AD have been considered in the literature. Potential prevention perspectives at both the individual and collective levels are also highlighted in this review. Future studies are encouraged in order to reach a consensus on accepted diagnostic criteria and to develop validated measures with a strong theoretical basis for the accurate diagnosis and treatment of AD.

## Figures and Tables

**Table 1:** Summary of the studies focusing on the treatment of Affective Dependence/Love Addiction.

Title	Year	Type of Study	Assessment Tool	Mentioned Intervention	References
Group therapy for pathological love	2008	Article	LAS	Psychodrama group therapy	[35]
Behavioural addictions: A novel challenge for psychopharmacology	2014	Narrative Review		Pharmacological treatment	[36]
Intense, Passionate, Romantic Love: A Natural Addiction? How the Fields That Investigate Romance and Substance Abuse Can Inform Each Other	2016	Narrative Review		“Healthy replacement reinforcers”	[37]
Addicted to love: What is love addiction and when should it be treated?	2017	Narrative Review		Professional counselling CBT Psychoanalysis Drug-based therapies	[38]
The Effect of Integrative Approach (Schema Therapy and Imago Therapy) on Girls’ Love Addiction in Isfahan	2018	Article	PALQ	Integrative approach (Imago therapy and schema therapy)	[39]
Treatment of love addiction: Current status and perspectives	2019	Narrative Review		Self-help groups (e.g., SLAA) CBT Psychodynamic Psychotherapy Psychodrama Group Psychotherapy Pharmacotherapy	[40]
Gestalt Therapy for Love Addiction.	2019	Article		Gestalt Therapy	[41]

Note: LAS: Love Attitudes Scale; PALQ: Peabody’s Addiction to Love Questionnaire; CBT: Cognitive Behavioural Therapy.

## Data Availability

All extracted data are available upon request.

## Abbreviations

AD	Affective Dependence
ADS-9	Affective Dependence Scale
CBT	Cognitive Behavioural Therapy
DPD	Dependent Personality Disorder
DSM-5	Diagnostic and Statistical Manual of Mental Disorders Fifth Edition
GT	Gestalt Therapy
ICD	Impulse-Control Disorder
LA	Love Addiction
LAI	Love Addiction Inventory
LASA	Love Addiction Self-Assessment
LS	Love Scale
LAS	Love Attitudes Scale
OCD	Obsessive-Compulsive Disorder
PALQ	Peabody’s Addiction to Love Questionnaire
PRISMA-SCR	PRISMA Extension for Scoping Reviews
SLAA	Sex and Love Addicts Anonymous
SSRIs	Selective Serotonin Reuptake Inhibitors
